# Influence of screw density on thoracic kyphosis restoration in hypokyphotic adolescent idiopathic scoliosis

**DOI:** 10.1186/s12891-017-1877-6

**Published:** 2017-12-13

**Authors:** Ming Luo, Honghui Jiang, Wengang Wang, Ning Li, Mingkui Shen, Peng Li, Genzhong Xu, Lei Xia

**Affiliations:** 1 Institute of Spinal Deformity, the First Affiliated Hospital of Zhengzhou University, Zhengzhou, Henan 450052 People’s Republic of China; 2 Department of Orthopaedic Surgery, Central Hospital of Wuhan, Tongji Medical College, Huazhong University of Science & Technology, Wuhan, People’s Republic of China

**Keywords:** Adolescent idiopathic scoliosis, Hypokyphosis, Thoracic kyphosis, Screw density

## Abstract

**Background:**

Previous studies have reported that rod composition and diameter, as well as the correction technique are key factors associated with thoracic kyphosis (TK) restoration. However, few study has analyzed the correlation between screw density and TK restoration in hypokyphotic adolescent idiopathic scoliosis (AIS).

**Methods:**

Fifty-seven thoracic AIS patients with preoperative TK < 10° treated with all pedicle screw fixation with a minimum 2-year follow-up were recruited. Preoperative and postoperative radiographic measurements, and information of posterior instrumentation were reviewed. Pearson and Spearman correlation coefficient analysis were used to assess relationships between change in TK and number of variables. Then, the included patients were classified into two groups (Group 1: postoperative TK ≥ 20°; Group 2: postoperative TK < 20°) to evaluate the influence factors of TK restoration.

**Results:**

The average preoperative TK was 4.75°, which was significantly restored to 17.30° (*P* < 0.001). Significant correlations were found between change in TK and flexibility of major thoracic curve (*r* = 0.357, *P* = 0.006), preoperative TK (*r* = −0.408, *P* = 0.002), and screw density of concave side (*r* = 0.306, *P* = 0.021), respectively. In the subgroup comparison, 17 patients (29.8%) maintain the postoperative TK ≥ 20°, increased flexibility of major thoracic curve (*P* < 0.001), screw number of concave side (*P* = 0. 029), and cobalt chromium rods (*P* = 0.041) were found in the group of postoperative TK ≥ 20°.

**Conclusions:**

TK restoration remains a challenge for AIS patients with hypokyphosis, especially for the poor flexibility ones. Except for thicker and cobalt chromium rods, screw density of concave side might be another positive predictor of restoring normal kyphosis, which provides a stronger corrective force on the sagittal plane with more pedicle screws.

## Background

Adolescent idiopathic scoliosis (AIS) is a tridimensional deformity afflicting millions of children who are at risk between the ages of 10–16 years. AIS has a prevalence of 2–4% in this population. The defined threshold for surgical treatment is when the major curve’s Cobb angle is greater than 40°. Untreated AIS patients may have pulmonary limitations, back pain, and changes in appearance and overall motor function [1].

Compared to the coronal correction, sagittal alignment restoration has received more attention [2–4]. Hypokyphosis, also called flat back, is one of the prominent features of AIS and has been defined as a sagittal curve (T5–T12) less than 10°, measured from the superior end-plate of the fifth thoracic vertebra to the inferior end-plate of the twelfth thoracic vertebra in the sagittal plane [5, 6]. Hypokyphosis has an adverse impact on pulmonary function and lumbar disc degeneration [7–10]. In addition, preservation of thoracic kyphosis (TK) is critical to maintain sagittal balance in the surgical treatment of AIS [2, 11, 12].

A Significant correlation between implant density and coronal correction has been previously observed in the surgical treatment of AIS [13–15]. However, few studies have evaluated the correlation between implant density and sagittal correction, and different conclusions were reported [16–18]. Larson et al. reported that increased implant density resulted in decreased TK in Lenke type 1 and 2 curves [16]. Conversely, Liu et al. demonstrated that high screw density on the concave side could provide better TK restoration [17]. Sudo et al. evaluated 36 AIS patients with preoperative TK with an angle less than 15° and found that screw density was an independently predictive of change in TK [18].

Previous studies have reported that rod composition and diameter, as well as the correction technique are key factors associated with TK restoration [19–23]. To our limited knowledge, few study has analyzed the correlation between TK restoration and screw density in hypokyphotic AIS (TK < 10°). The purpose of the study is to evaluate the influence factors of TK restoration, especially for screw density, rod composition and diameter, which associated with all pedicle screw fixation.

## Methods

### Patients identification

After receiving approval from the institutional review board, the operative and radiological notes of consecutive AIS patients were retrospectively reviewed. The patients that were reviewed underwent spinal surgery from January 2009 to December 2014 at a single institution.

Selection criteria included the following: (1) Lenke type 1–4 AIS; (2) preoperative TK (T5–T12) < 10°; (3) one-stage posterior approach; (4) all pedicle screw construct, and (5) minimum 2-year follow-up. Exclusion criteria included the following: (1) Lenke type 5–6 AIS, early-onset scoliosis, or neuromuscular scoliosis; (2) hybrid instrumentation constituted with hooks or wires; (3) anterior–posterior approach; (4) pedicle subtraction osteotomy or vertebral column resection performed; and (5) reoperations related to the pedicle screw system.

### Surgical technique

Two senior surgeons performed all the surgeries. After the standard posterior midline incision and the anatomical exposure of the spine, multilevel inferior facet resection and superior facet decortication were undergoing. After pedicle screws were implanted with a free hand technique, a metal rod was bended to the anticipated TK prior to insertion, and rod reduction, in order to turn the locking cap to reduce the rod into the screw head, were performed after the insertion of the concave rod. Simple rod rotation technique was applied for deformity correction. After insertion of the second rod and tightening of locking caps, distraction and compression were performed, and locking caps were finally tightened. Transverse connector was selectively used. The transverse processes and laminae were decorticated thoroughly, and allograft bone material was placed for fusion.

### Clinical and radiographic parameters

Clinical measurements included the patients’ age at surgery, gender, rod diameter, rod material, fused vertebral levels, and number of pedicle screws that were used. Screw density was defined as the number of pedicle screws per vertebrae that were implanted. Concave side screw density was calculated using the screw number of the concave side divided by the fused levels; convex side screw density was calculated in a same manner. Total screw density was calculated using the total screw number divided by the fused levels.

Standing posterior-anterior and lateral radiographs were measured for parameters before the surgery and at the last follow-up. Radiographic measurements included the patients’ Lenke classification, Risser grade, apical vertebral rotation (Nash-Moe), Cobb angle of main thoracic (MT) curve, convex bending, TK (T5–T12), and lumbar lordosis (T12–S1). The flexibility index, MT correction rate, change in MT curve, and change in TK (postoperative TK – preoperative TK) were all calculated from above radiographic parameters.

### Statistical analyses

In order to explore the correlation between change in TK and potential variables, including age, flexibility of MT curve, preoperative MT Cobb angle, preoperative TK, preoperative LL, fused levels, screw density of concave side, screw density of convex side, and total screw density, Pearson and Spearman correlation coefficient analysis were performed. According to the Lenke classification, the N of the sagittal modifier is from 10° to 40°. However, many scholars hold the view that postoperation TK (T5-T12) less than 20° is still hypokyphosis with, and it is generally agreed that operative treatment of thoracic idiopathic scoliosis should aim to improve TK to a degree more than 20° [7, 24]. In order to evaluate the influence factors of a satisfied TK restoration, the cohort was then divided into two groups based on postoperative TK with the threshold of 20°. All data were collected and analyzed using IBM SPSS Statistics v.24.0 (IBM Corp., Armonk, N.Y., USA). *P* < 0.05 was considered statistically significant.

## Results

Fifty-seven consecutive thoracic AIS patients with hypokyphosis were carefully reviewed. There were 45 females and 12 males, and the average age at surgery was 14.39 ± 1.82 (range, 12–19) years old. According to the Lenke classification, [6] there were 28 type 1, 16 type 2, 13 type 3 curves. Preoperative radiographs showed an average MT curve of 55.23° ± 11.65° (range, 37°–91°), and the average preoperative TK was 4.75° ± 3.45° (range, −4°–9°). Postoperative MT significantly reduced to 15.74° ± 7.64° (range, 2°–35°), and postoperative TK was significantly restored to 17.30° ± 5.13° (range, 7°–30°).

**Fig. 1:**
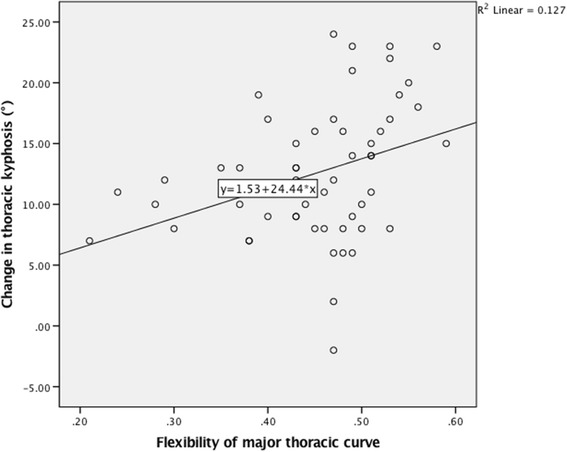
Correlation between change in thoracic kyphosis and flexibility of MT curve

**Fig. 2:**
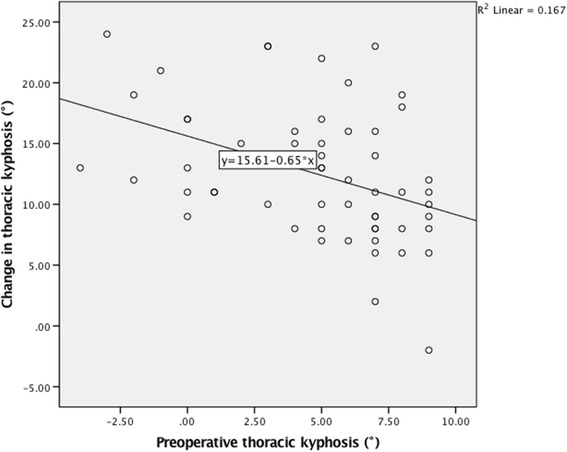
Correlation between change in thoracic kyphosis and preoperative thoracic kyphosis

**Fig. 3:**
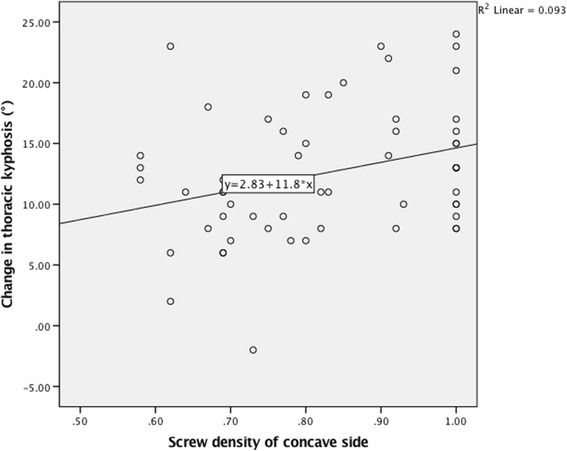
Correlation between change in thoracic kyphosis and convex side screw density

In the correlation analysis between change in TK and variables, a linear trend was found between change in TK and flexibility of MT curve (Pearson: *r* = 0.357, *P* = 0.006; Spearman: r_s_ = 0.384, *P* = 0.003. Figure 1). A linear trend was also found between change in TK and preoperative TK (Pearson: *r* = −0.408, *P* = 0.002; Spearman: r_s_ = −0.445, *P* = 0.001. Figure 2). In addition, a positive correlation was found between change in TK and concave side screw density (Pearson: *r* = 0.306, *P* = 0.021; Spearman: r_s_ = 0.290, *P* = 0.029. Figure 3). The correlation analysis between change in TK and variables are presented in Table 1.

**Table 1:** Correlation analysis between change in thoracic kyphosis and variables

Variable	Pearson correlation coefficients			Spearman correlation coefficients		
	Correlation coefficient	95% CI	Statistical significance	Correlation coefficient	95% CI	Statistical significance
Age	−0.025	(−0.217, 0.178)	0.853	0.017	(−0.220, 0.269)	0.899
Flexibility (%)	0.357	(0.171, 0.534)	0.006	0.384	(0.136, 0.578)	0.003
Preoperative MT Cobb angle (°)	−0.138	(−0.390, 0.136)	0.304	−0.097	(−0.369, 0.180)	0.475
Preoperative TK (T5–T12; °)	−0.408	(−0.609, −0.186)	0.002	−0.445	(−0.626, −0.214)	0.001
Preoperative LL (L1–S1; °)	0.148	(−0.152, 0.459)	0.272	0.237	(−0.022, 0.476)	0.076
Fused levels	0.010	(−0.207, 0.220)	0.941	0.002	(−0.254, 0.267)	0.987
Screw density of concave side	0.306	(0.052, 0.502)	0.021	0.290	(0.016, 0.513)	0.029
Screw density of convex side	0.061	(−0.222, 0.329)	0.655	0.063	(−0.205, 0.318)	0.643
Total screw density	0.206	(−0.069, 0.427)	0.123	0.213	(−0.054, 0.441)	0.112

MT indicates main thoracic; *TK* thoracic kyphosis, *LL* lumbar lordosis

In the subgroup analysis, the postoperative TK ≥ 20°group consisted of 17 patients (29.8%; 5.5 mm Ti:6.0 mm Ti:5.5 mm CoCr:6.0 mm CoCr = 0:7:4:6), whereas the postoperative TK < 20°group had 40 patients (70.2%; 5.5 mm Ti:6.0 mm Ti:5.5 mm CoCr:6.0 mm CoCr = 5:23:10:2). No significant differences were found with respect to the following parameters: gender, age at surgery, Riser sign, apical vertebra rotation, convex-Bending Cobb angle, preoperative MT Cobb angle, postoperative MT Cobb angle, change in MT Cobb angle, MT curve correction, preoperative TK, preoperative LL, postoperative LL, change in LL, rod diameter (5.5 mm/6.0 mm), fused levels, total screw number, screw number of convex side, total screw density, screw density of concave side, screw density of convex side. Except for significant differences in postoperative TK (*P* < 0.001) and change in TK (*P* < 0.001), poor flexibility of MT curve (*P* < 0.001), lower incidence rate of CoCr rod (*P* = 0.041), and smaller screw number of concave side (*P* = 0.029) were found in the postoperative TK < 20°group. Interestingly, the MT curve correction (%) in the postoperative TK ≥20° group was six percentage points lower than the other group, even though no significant difference was found (68 ± 13 vs 74 ± 10, *P* = 0.079). The clinical parameters are presented in Table 2 and the radiographic parameters are showed in Table 3.

**Table 2:** Subgroup comparison of clinical parameters

Variable	Group 1 (Postop TK ≥ 20°)	Group 2 (Postop TK<20°)	*P* value
Gender (female/male)	13/4	32/8	0.765
Age at surgery (year)	14.41 ± 1.50	14.38 ± 1.96	0.945
Rod diameter (5.5 mm/6.0 mm)	4/13	15/25	0.306
Rod material (Ti/CoCr)	7/10	28/12	0.041
Fused levels	12.18 ± 1.33	11.38 ± 1.69	0.088
Total screw number	18.06 ± 3.82	16.43 ± 3.53	0.124
Screw number of concave side	10.35 ± 1.73	9.15 ± 1.90	0.029
Screw number of convex side	7.71 ± 2.37	7.28 ± 2.03	0.488
Total screw density	1.49 ± 0.28	1.45 ± 0.25	0.637
Screw density of concave side	0.85 ± 0.13	0.81 ± 0.14	0.279
Screw density of convex side	0.63 ± 0.18	0.64 ± 0.15	0.854

Values are mean ± standard deviation, number of participants, or as otherwise indicated

Ti indicates titanium, *CoCr* cobalt chromium

**Table 3:** Subgroup comparison of radiographic parameters

Variable	Group 1 (Postop TK ≥ 20°)	Group 2 (Postop TK<20°)	*P* value
Lenke classification (1/2/3)	10/4/3	18/12/10	–
Riser sign	2.47 ± 1.23	2.90 ± 1.46	0.294
Apical vertebra rotation (Nash-Moe)	1.71 ± 0.47	1.60 ± 0.67	0.558
Convex-Bending Cobb angle (°)	27.12 ± 5.48	31.53 ± 7.33	0.804
Flexibility (%)	51 ± 4	42 ± 8	<0.0001
Preoperative MT Cobb angle (°)	55.82 ± 10.48	54.98 ± 12.23	0.804
Postoperative MT Cobb angle (°)	17.88 ± 7.53	14.83 ± 7.59	0.169
Change in MT Cobb angle (°)	37.94 ± 9.65	40.15 ± 8.28	0.384
MT curve correction (%)	68 ± 13	74 ± 10	0.079
Preoperative TK (T5–T12; °)	5.18 ± 3.28	4.58 ± 3.54	0.552
Postoperative TK (T5–T12; °)	23.41 ± 3.20	14.70 ± 3.22	<0.0001
Change in TK (T5–T12; °)	18.24 ± 4.05	10.13 ± 3.99	<0.0001
Preoperative LL (L1–S1; °)	−45.76 ± 9.68	−48.80 ± 9.33	0.271
Postoperative LL (L1–S1; °)	−50.82 ± 8.75	−51.35 ± 8.49	0.833
Change in LL (L1–S1; °)	−5.06 ± 13.51	−2.55 ± 10.35	0.449

Values are mean ± standard deviation, number of participants, or as otherwise indicated

MT indicates main thoracic; *TK* thoracic kyphosis, *LL* lumbar lordosis

## Discussion

Increased attention should be allocated to TK restoration, as it remains a challenge for the surgical treatment of thoracic AIS patients. Decreases in TK after posterior spinal fusion using segmental pedicle screw construct has gained increased attention [2, 25, 26]. Although there is insufficient evidence demonstrating that hypokyphosis affects the clinical outcome in AIS patients, [27] directly or indirectly, influences on pulmonary function, [8, 9, 28] adjacent-segment disease, [29] cervical sagittal alignment, [30] and lumbar lordosis have been found to be related to a decrease in TK after surgical treatment [31].

TK restoration is a continuing surgical challenge for AIS patients with hypokyphosis [21]. In our study, only 29.8% hypokyphotic AIS achieved satisficed TK restoration (postoperative TK ≥ 20°). A positive correlation was found between change in TK and flexibility of MT curve, and in the subgroup analysis, poor flexibility of MT curve was found in the postoperative TK < 20°group, which suggested that TK restoration was more difficult for patients with a poor flexibility. Interestingly, that was, in our series, if more MT curve correction was achieved, less restoration of TK occurred. It was suggested that excessive correction of the coronal plane was at the expense of sagittal contour. Quan et al. firstly reported that postoperative TK change was negatively correlated with magnitude of coronal Cobb angle correction (*P* = 0.002), and the mechanism was unclear [32]. To our limited knowledge, this might be due to the distraction manipulation of the concave rod, which was aimed at a better coronal correction, unexpectedly consumed the bended rod contour, and further biomechanics study is needed.

The most commonly studied factors influencing TK restoration include the implant rod stiffness, [17, 33] implant rod curvature, [19, 34] anterior endoscopic release, [22] and simultaneous double-rod rotation technique [23, 35, 36]. As regard to implant rod stiffness, lower incidence rate of CoCr rod (*P* = 0.029) were found in the postoperative TK < 20°group in our study. A biomechanical study reported that the correctional force produced by the Ti 30-degree pre-bend rod was approximately 67% that of a CoCr rod [37]. Similar studies also supported using CoCr rod in hypokyphotic AIS patients [38, 39]. However, no significant difference was found in rod diameter (5.5 mm/6.0 mm) in the subgroup analysis, the relatively small sample size and short follow-up time might the two reasons, and a longer follow-up study should be performed to assess the maintenance of TK restoration. In addition, removing the posterior elements (Ponte osteotomy or facetectomy) might increase flexibility and allow the posterior column to lengthen [40]. Shah et al. found a significant restoration in TK from 8.1° to 18.3° (*P* < 0.001) in hypokyphotic curves treated with Ponte osteotomy [41]. Sudo et al. reported a significant correlation between change in TK and number of facetectomy level (*r* = 0.492, *P* = 0.002) [42]. Therefore, CoCr rod and posterior column release should be taken into consideration for hypokyphotic AIS.

Few studies have evaluated the correlation between implant density and sagittal correction, and conclusions were controversial. Liu et al. compared the effects of high versus low implant density on sagittal plane correction, and greater TK restoration was found in AIS patients with high screw density on the concave side (*P* < 0.05) [17]. Similarity, Sudo et al. retrospectively reviewed Lenke type 1 AIS patients with preoperative TK < 15°, and change in TK was significantly correlated with concave side screw density (*r* = 0.351) but not with convex side screw density (*r* = 0.144) [18]. Contrarily, Larson et al. retrospectively reviewed a multicenter database and found decreased TK (T2–T12) with an increased implant density for Lenke type 1 (*n* = 375) and type 2 (*n* = 245) curves [16]. While, the included patients were not classified into hypokyphosis, normal kyphosis, and hyperkyphosis based on TK. The difference between the average angles of TK was probably the primary cause for differing conclusions.

Through this study, we found that concave side screw density was positively correlated with TK restoration in thoracic AIS patients with hypokyphosis, In addition, smaller screw number of concave side were found in the postoperative TK < 20°group. Which suggested that higher concave side screw density provided a beneficial effect on TK restoration in hypokyphotic AIS. A possible explanation is that the concave side rod provides a stronger corrective force on the sagittal plane with more pedicle screws. Cidambi et al. reported that the resulting deformations were likely to be associated with substantial in vivo deforming forces, particularly for concave rods [20]. Similar conclusion were demonstrated by Salmingo et al. [19]. In addition, Abe et al. analyzed scoliosis corrective forces and pull out forces based on finite element analysis and found that the corrective force was roughly four times greater in the concave side than in the convex side [43]. Implanted pedicle screws were under the effect of two forces that included the corrective force exerted from the stiffness of the rod and the resistant force from the deformed spine. It was possible that more pedicle screws were inserted at the concave side rod, providing a stronger pullout force. According to reciprocity of force, the stronger pullout force would convert into a corrective force and could be evenly applied on the hypokyphosis spine. Then, the hypokyphosis spine would be restored and follow the curvature of the concave side rod.

An important limitation of the study was that the analysis of rod curvature prior to implantation was limited due to the retrospective study. In addition, the relatively small sample size and short follow-up time were underpowered to find more significant difference, and larger-scale and longer follow-up research should be performed.

## Conclusions

TK restoration remains a challenge for AIS patients with hypokyphosis, especially for poor flexibility ones. Except for thicker and cobalt chromium rods, screw density of concave side might be another positive predictor of restoring normal kyphosis, which provides a stronger corrective force on the sagittal plane with more pedicle screws.

## Abbreviations

AIS: Adolescent idiopathic scoliosis
MT: Main thoracic
TK: Thoracic kyphosis
